# The Effector TepP Mediates Recruitment and Activation of Phosphoinositide 3-Kinase on Early *Chlamydia trachomatis* Vacuoles

**DOI:** 10.1128/mSphere.00207-17

**Published:** 2017-07-19

**Authors:** Victoria Carpenter, Yi-Shan Chen, Lee Dolat, Raphael H. Valdivia

**Affiliations:** Department of Molecular Genetics and Microbiology and Center for Host-Microbe Interactions, Duke University School of Medicine, Durham, North Carolina; Carnegie Mellon University

**Keywords:** CrkL, PI3K, PIP3, Src kinases, T3S effector, type I IFN, effector proteins

## Abstract

*Chlamydia trachomatis* delivers multiple type 3 secreted effector proteins to host epithelial cells to manipulate cytoskeletal functions, membrane dynamics, and signaling pathways. TepP is the most abundant effector protein secreted early in infection, but its molecular function is poorly understood. In this report, we provide evidence that TepP is important for bacterial replication in cervical epithelial cells, activation of type I IFN genes, and recruitment of class I phosphoinositide 3-kinases (PI3K) and signaling adaptor protein CrkL to nascent pathogen-containing vacuoles (inclusions). We also show that TepP is a target of tyrosine phosphorylation by Src kinases but that these modifications do not appear to influence the recruitment of PI3K or CrkL. The translocation of TepP correlated with an increase in the intracellular pools of phosphoinositide-(3,4,5)-triphosphate but not the activation of the prosurvival kinase Akt, suggesting that TepP-mediated activation of PI3K is spatially restricted to early inclusions. Furthermore, we linked PI3K activity to the dampening of transcription of type I interferon (IFN)-induced genes early in infection. Overall, these findings indicate that TepP can modulate cell signaling and, potentially, membrane trafficking events by spatially restricted activation of PI3K.

**IMPORTANCE** This article shows that *Chlamydia* recruits PI3K, an enzyme important for host cell survival and internal membrane functions, to the pathogens inside cells by secreting a scaffolding protein called TepP. TepP enhances *Chlamydia* replication and dampens the activation of immune responses.

## Abstract (precis)

This article shows that chlamydia recruit PI3K, an enzyme important for host cell survival and internal membrane functions, to the pathogens inside cells by secreting a scaffolding protein called TepP. TepP enhances *Chlamydia* replication and dampens the activation of immune responses.

## INTRODUCTION

*Chlamydia trachomatis* is an obligate intracellular bacterial pathogen of significant socioeconomic and medical importance. *C. trachomatis* is the leading causative agent of preventative blindness worldwide and the most prevalent sexually transmitted infection (STI) in the Western world (1, 2). *C. trachomatis* undergoes two main developmental transitions, with an infectious form, the elementary body (EB), and a replicative form, the reticulate body (RB). Both the RB and EB forms of the pathogen manipulate host cellular functions by delivering type 3 secretion (T3S) effector proteins directly into the membranes and cytoplasm of target cells (3).

The *Chlamydia* T3S system shares many functional features with T3S systems from other Gram-negative bacteria, including the requirement for accessory chaperones that stabilize effectors and enhance their secretion (3–5). One of these T3S chaperones, Slc1, interacts with and enhances the secretion of multiple EB effectors (6). For instance, the effector Tarp (translocated actin recruiting phosphoprotein) is delivered into epithelial cells within 5 min of EB attachment and phosphorylated at tyrosine residues (7–9). Multiple proteins with Src homology 2 (SH2) domains can bind *in vitro* to peptides representing the phosphorylated forms of Tarp (10, 11). These include the E3 ligase Cbl; the Rac1 exchange factor Vav2; the p85 regulatory subunit of phosphoinositide 3-kinase (PI3K); the signaling adaptors Shc1, Nck2, and CrkL; and the kinase Syk (12). Various tyrosine kinases can phosphorylate Tarp *in vitro* and *in vivo*, including Src, Abl, and Syk (7, 8). Tarp nucleates F-actin assembly, independently of its tyrosine phosphorylation status, through its F-actin-binding domain and interaction with the Arp2/3 complex, and by activating focal adhesion kinases (FAK) through molecular mimicry of the cofactor paxillin (9, 11, 13–16). Not surprisingly, this multifunctional effector is important for *Chlamydia* infection, as microinjection of anti-Tarp antibodies into epithelial cells or expression of dominant-negative Tarp constructs in *Chlamydia* inhibits bacterial invasion (11, 17).

A second Slc1-dependent effector is the translocated early phospho-protein (TepP). On a molar basis, TepP is one of the most abundant *Chlamydia*-specific proteins found within EBs (18). Once translocated into the host cell, TepP is phosphorylated by host cell kinases at multiple tyrosine and serine residues (6). Two tyrosine phosphorylation (pY) sites generate consensus pYxxP motifs that provide a docking site for proteins with SH2 domains, including two spliced forms of the signaling adaptor protein Crk (Crk I and Crk II [Crk I/II]) (reviewed in reference 19). Indeed, CrkI and CrkII coimmunoprecipitate with TepP and are recruited to nascent *C. trachomatis* inclusions in a TepP-dependent manner (6). A comparison of the transcriptional responses of epithelial cells to infection with a *tepP*-deficient mutant and TepP-overexpressing strains indicated a role for TepP in the induction of a subset of genes associated with type I interferon (IFN) responses, including interferon-induced peptides with tetratricopeptide repeats (*IFIT* [20]) (6).

To address the mechanism by which TepP modulates host cellular functions, we identified host proteins that associate with TepP during the early stages of bacterial invasion and establishment of inclusions. We determined that CrkL and class I phosphoinositide 3-kinases (PI3K) (21) are the major proteins that copurify with TepP and that these proteins are recruited to nascent inclusion in a TepP-dependent manner. Furthermore, TepP induces the activation of PI3K on internal membranes and nascent inclusions to generate phosphoinositide-(3,4,5)-triphosphate (PIP3) without activating canonical PI3K signaling at the plasma membrane.

## RESULTS

### CrkL and PI3K copurify with TepP translocated during infection.

**FIG 1:**
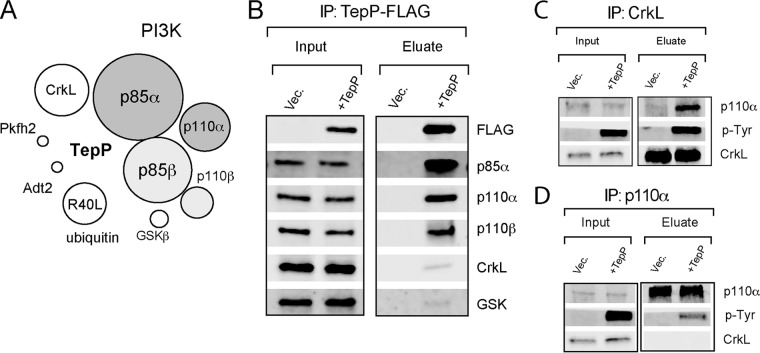
TepP forms complexes with PI3K and CrkL in infected cells. (A) Schematic representation of epithelial proteins that associate with TepP during infection. A2EN cells were infected with *C. trachomatis* expressing TepP-FLAG for 4 h, and cell lysates were subjected to immunoprecipitation (IP) with anti-FLAG antibodies. Bound proteins were digested with trypsin, and the resulting peptides were identified by mass spectrometry (MS). Sizes of circles correspond to the relative numbers of peptides identified for each protein by LC-MS/MS. (B) Immunoblot validation of TepP-interacting proteins. A2EN cells were infected for 4 h with CTLM062G1 transformed with a TepP-FLAG expression plasmid or vector-only control (Vec.), and cell lysates were subjected to IP with anti-FLAG antibodies. Bound proteins were detected by immunoblotting with specific antibodies. (C and D) Reciprocal co-IP of CrkL (C), PI3K (D), and TepP. A2EN cells were infected with CTLM062G1 strains as described for panel B, and at 4 h postinfection, CrkL and p110α were subjected to IP. Co-IP of proteins was assessed by immunoblot analysis.

TepP is phosphorylated at multiple tyrosine residues upon delivery into host cells (6) and may directly recruit Src homology 2 (SH2) and phosphotyrosine-binding (PTB) domain-containing proteins to assemble novel host cell signaling complexes (22, 23). To identify *Chlamydia* and host proteins associated with TepP-containing signaling complexes, we infected A2EN endocervical epithelial cells with *tepP* null mutant strain CTL2-M062G1 and with variants transformed with either an empty plasmid (pVec) or a plasmid expressing TepP-FLAG (pTepP). After 4 h, infected cells were lysed under nondenaturing conditions and subjected to immunoprecipitation (IP) with anti-FLAG antibodies. All proteins in the IP were digested with trypsin and the resulting peptides identified by liquid chromatography coupled to tandem mass spectrometry (LC-MS/MS). The major human proteins copurifying exclusively with TepP-FLAG included the catalytic (p110α and p110β) and regulatory (p85α and p85β) subunits of PI3K, CrkL, and glycogen synthase kinase (GSK) (Fig. 1A; see also Table S1 in the supplemental material). The specificity of these interactions was verified by immunoblot analysis of subsequent IPs. CrkL, GSK, and both PI3K subunits (p110 and p85) coprecipitated with TepP during infection (Fig. 1B). Reciprocal IP of CrkL and p110α coprecipitated phospho-TepP from infected cells (Fig. 1C and D), validating the specificity of these interactions.

10.1128/mSphere.00207-17.4 TABLE S1  Summary of proteins identified by LC-MS/MS as potential binding partners for TepP. Download TABLE S1, XLSX file, 0.03 MB. Copyright © 2017 Carpenter et al. 2017 Carpenter et al. This content is distributed under the terms of the Creative Commons Attribution 4.0 International license.

### TepP-deficient mutants fail to recruit PI3K and CrkL to early inclusions.

**FIG 2:**
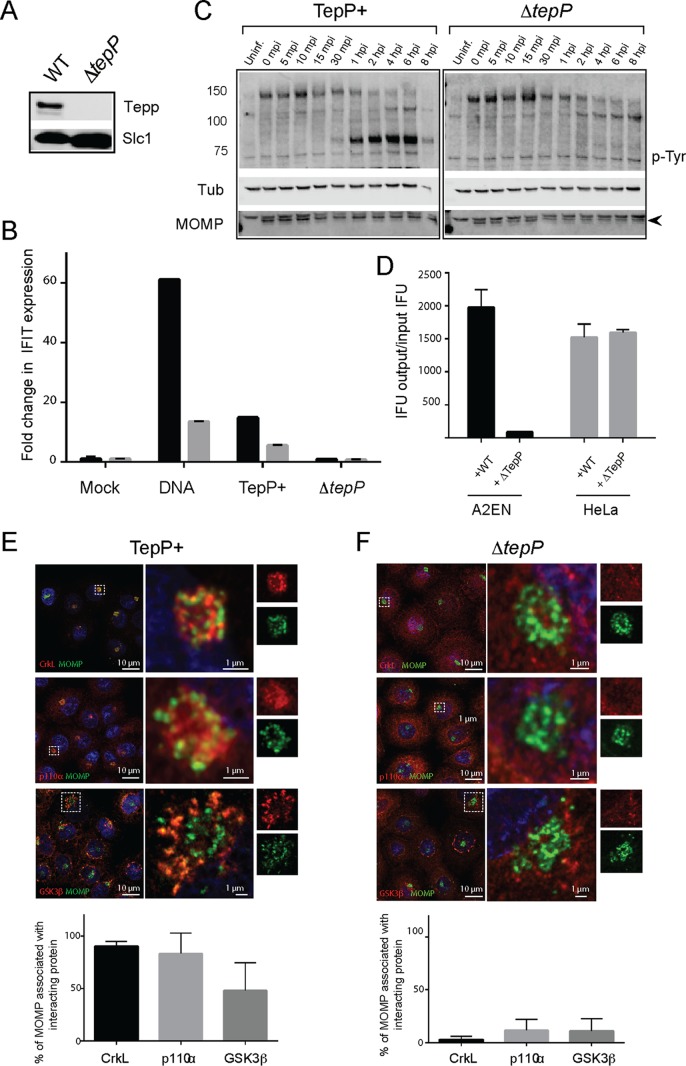
TepP is required for *C. trachomatis* replication in A2EN cells and to recruit CrkL and PI3K to nascent inclusions. (A to D) Phenotypic characterization of a *C. trachomatis* Δ*tepP*::*bla* insertional mutant. (A) Immunoblot analysis of TepP expression in HeLa cells infected for 24 h with the indicated strains. Slc1, loading control. (B) A2EN cells were infected with CTL2 or Δ*tepP*::*bla* bacteria, and the levels of *IFIT1* (black) and *IFIT2* (gray) transcripts from the strains described in the panel A legend were assessed by quantitative RT-PCR. DNA transfection was used as a positive control for the induction of *IFIT* genes. (C) Time course of appearance of major tyrosine-phosphorylated (p-Tyr) proteins in A2EN cell infected with CTL2 or Δ*tepP*::*bla* insertional mutants or left uninfected (Uninf.). MOMP, bacterial major outer membrane protein (arrowhead); Tub, tubulin. (D) The replication potential of Δ*tepP*::*bla* mutants was assessed in HeLa and A2EN cells by the generation of inclusion-forming units (IFU). (E and F) Subcellular localization of CrkL, PI3K (p110α), and GSK-3β in *Chlamydia*-infected cells. A2EN cells were infected with CTL2 or Δ*tepP*::*bla* bacteria for 4 h and immunostained by anti-MOMP (green), anti-CrkL, anti-p110α, or anti-GSK-3β (red). Hoechst (blue) was used to detect DNA. Quantification of *Chlamydia* (MOMP) and p110α, CrkL, and GSK-3β colocalization was performed on a single-cell basis (*n =* 20 and 30 cells/replicate). Student’s *t* test showed that there was a statistically significant difference between CTL2 and Δ*tepP*::*bla* bacteria, with a *P* value of <0.001.

To perform a clean phenotypic analysis of TepP mutants, we disrupted the *tepP* gene in CTL2 with a gene encoding β lactam resistance (*bla*) by group II intron-mediated gene insertion (TargeTron) (24). TepP could not be detected in insertional mutants by immunoblot analysis (Fig. 2A). The resulting *tepP*::*bla* (*ΔtepP*) strain behaved like the CTL2-M062G1 *tepP* nonsense mutant in that it failed to induce the expression of interferon-induced peptides with the tetratricopeptide repeat (*IFIT*) genes (Fig. 2B) that are typically activated during the early stages of infection (6) and blocked the tyrosine phosphorylation of a subset of host proteins (Fig. 2C) (6). We next determined if TepP is important for efficient bacterial replication by comparing the results of generation of infectious particles in cell lines infected with either the wild-type (WT) strain or Δ*tepP* mutants. A2EN cells, representing a newly derived cervical epithelial cell line (25), were particularly restrictive for the replication of *ΔtepP* stains compared to HeLa cells (Fig. 2D). This defect appears to have been unrelated to invasion, as Δ*tepP* mutants were not impaired for entry into cells (data not shown).

We next tested if PI3K and CrkL are recruited to the early inclusions where TepP translocation is most apparent (6). We infected A2EN cells with wild-type or *ΔtepP C. trachomatis* and determined the extent of colocalization of TepP binding partners with intracellular bacteria by indirect immunofluorescence. Both PI3K (83%) and CrkL (90%) were strongly associated with clusters of intracellular bacteria that had migrated to a perinuclear region of host cells by 4 h postinfection (hpi) (Fig. 2E). The accumulation of GSK at early inclusions was less prominent (48%). In contrast, we observed very little association of PI3K, CrkL, or GSK with early inclusions formed by TepP-deficient *Chlamydia* (Fig. 2F). Similar observations were made in HeLa and mouse embryonic fibroblasts (MEFs) (data not shown). Because PI3K, Crk adaptors, and GSK are involved in multiple signaling pathways and can impact the organization of the cytoskeleton, activation of innate immune factors, and expression of prosurvival signals (19, 21, 26, 27), our findings suggest that TepP recruits these adaptor proteins to influence signaling events.

### Src family kinases mediate the tyrosine phosphorylation of TepP, but Src activity is not required for the recruitment of PI3K and CrkL to early inclusions.

**FIG 3:**
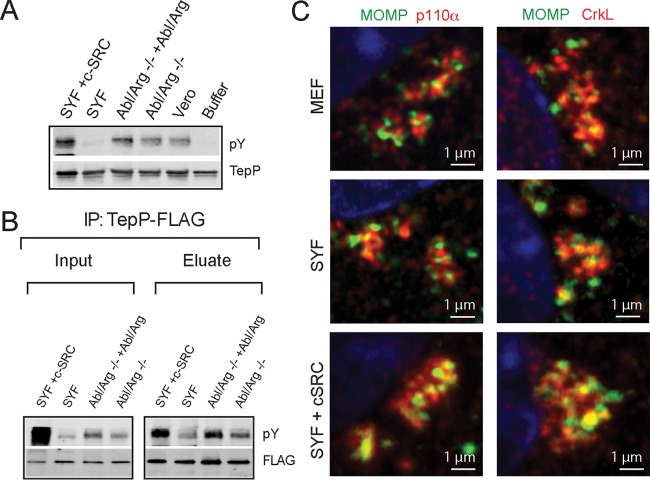
TepP is phosphorylated by Src kinases. (A) *In vitro* phosphorylation reactions were performed by incubating recombinant 6×His-TepP with ATP and cell lysates derived from the indicated cell lines. The degree of TepP phosphorylation was assessed by immunoblotting with anti-pY antibodies after reisolation of TepP on nickel beads. TepP was phosphorylated under all conditions except after incubation with SYF cell lysates and the buffer-only control. (B) TepP-FLAG immunoprecipitations from cell lines infected with CTLM062G1 expressing TepP-FLAG. TepP phosphorylation was assessed after IP with anti-FLAG antibodies and immunoblotting with anti-pY antibodies. Note the decreased levels of phosphorylation in Src, Yes, and Fyn-deficient cells. (C) Mouse embryo fibroblast (MEF), SYF, and SYF-plus-c-Src cell lines were infected for 4 h with *C. trachomatis* at an MOI of 20, fixed, and stained. Infected cells were immunostained by anti-MOMP (green), anti-CrkL, or anti-p110α (red) antibodies.

Because Tarp is phosphorylated by Src and Abl kinases (7, 8), we next tested if these kinases also play a role in the phosphorylation of TepP (7, 8, 28) by performing *in vitro* phosphorylation assays. Hexahistidine-tagged TepP purified from *Escherichia coli* was incubated with ATP and cytosolic extracts derived from the following: Vero cells, Abl^−^/Arg^−^ MEFs, Src/Yes/Fyn-deficient (SYF) MEFs, and their rescued counterparts. TepP was reisolated and the extent of tyrosine phosphorylation assessed by immunoblot analysis with anti-phosphotyrosine antibodies. Recombinant TepP was phosphorylated after incubation with all cell lysates except for those derived from SYF MEFs, suggesting that Src family kinases are required for TepP tyrosine phosphorylation *in vitro* (Fig. 3A). To determine if Src performs a similar function *in vivo*, the same cells lines were infected with CTL2-M062G1 expressing TepP-FLAG. After IP with anti-FLAG antibodies, we assessed the extent of TepP tyrosine phosphorylation by immunoblot analysis (Fig. 3B). We observed diminished phosphorylation of TepP during infection of SYF MEFs and increased phosphorylation upon overexpression of c-Src and, to a lesser extent, of Abl and Arg. Similar defects in TepP phosphorylation were observed in A2EN cells incubated with Src inhibitors (not shown). Taken together, these findings suggest that the Src family kinases phosphorylate TepP during infection, although other kinases may also play an auxiliary role.

We hypothesized that Src-mediated phosphorylation of TepP would regulate the recruitment of SH2 and PTB domain proteins like PI3K (p85 subunit) and CrkL. We infected SYF and SYF-plus-Src MEFs with wild-type *C. trachomatis* and assessed the recruitment of PI3K and CrkL to nascent inclusions by immunofluorescence microscopy. Surprisingly, Src kinases were not required for the recruitment of PI3K or CrkL to inclusions (Fig. 3C). As a complementary approach, we generated phenylalanine substitutions at the three tyrosines (Y43, Y496, and Y504) identified as TepP phosphorylation sites (6). The TepP^Y43F/Y496F/Y504F^ variant was still capable of pulling down PI3K and CrkL, and the mutant strain recruited these factors to early inclusions. However, this TepP variant was still tyrosine phosphorylated during infection (see Fig. S1 in the supplemental material), suggesting that additional tyrosine-phosphorylated residues in TepP may mediate interactions with host proteins.

10.1128/mSphere.00207-17.2 FIG S1  The TepP^Y43F/Y496F/Y504F^ mutant is tyrosine phosphorylated and capable of recruiting CrkL and p110α to early inclusions. (A) A2EN cells were infected with CTL2-M062G1 (pVec), CTL2-M062G1 (pTepP), and CTL2-M062G1 (pTepP^Y43F/Y496F/Y504F^) for 1, 2, and 4 h. Results of immunoblot analyses performed with anti-pY and anti-FLAG indicate levels of recombinant protein expression and tyrosine phosphorylation. (B) A2EN cells were infected with CTL2-M062G1 (pTepP^Y43F/Y496F/Y504F^) for 4 h, fixed, and immunostained for *Chlamydia* with anti-MOMP antibodies (green). CrkL and p110α (red) were detected with specific antibodies. Nucleic acids were stained with Hoechst (blue). Download FIG S1, TIF file, 1.1 MB. Copyright © 2017 Carpenter et al. 2017 Carpenter et al. This content is distributed under the terms of the Creative Commons Attribution 4.0 International license.

Taken together, these data suggest that while Src kinases are important for the full phosphorylation of TepP, these modifications do not appear to be an essential determinant for the interaction of TepP with PI3K or CrkL in infected cells.

### PI3K association with TepP at early inclusions is independent of CrkL.

**FIG 4:**
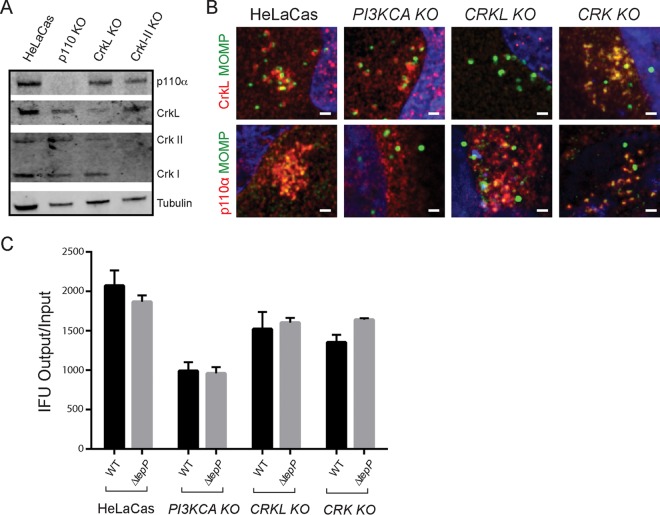
PI3K and CrkL independently associate with early inclusions. (A) HeLa cells stably expressing Cas9 (HeLa-Cas) were transduced with sgRNAs specific to *PIK3KCA*, *CRKL*, and *CRKI-II* to generate gene-edited cell lines lacking expression of the respective target proteins. Loss of protein expression for each cell line was verified by immunoblot analysis using anti-p110α, anti-CrkL, and anti-Crk I/II antibodies. Tubulin levels were used as positive controls. There was a low level of cross-reactivity between CrkL and CrkII antibodies. (B) CrkL and p110α are recruited to early inclusions formed in *PI3KCA* and *CRKL* gene-edited knockout cells (KO), respectively. HeLa-Cas cells and their edited derivatives were infected with CTL2 for 4 h, fixed, and immunostained with anti-MOMP and anti-p110α or anti-CrkL (red) antibodies. Host and bacterial DNA were detected with Hoechst (blue). (C) Replication of WT and TepP-deficient *C. trachomatis* in *PI3KCA*, *CRKL*, and *CRK* gene-edited knockout cells (KO).

PI3K and Crk adaptors are important signal transducers in growth factor-mediated activation of receptor tyrosine kinases (29, 30). Both p85 and CrkL have SH3 and SH2 domains that mediate protein-protein interactions. Proteins that bind to Crk proteins at both the SH2 and SH3 domains include p85 itself, paxillin, C3G, Abl, Arg, Cas, and Sos (19, 22, 30–32). Similarly, the p110 and p85 PI3K subunits interact with a variety of kinases, including Fyn, Lyn, Src, Fak, and Bcr (33, 34). Because CrkL and PI3K can interact with each other (19, 22, 31) we tested if the association of these proteins with TepP is cooperative. We generated HeLa cell lines lacking the p110α subunit of PI3K, CrkL, or CrkI/II by clustered regularly interspaced short palindromic repeat (CRISPR)/Cas9-mediated gene disruption (Fig. 4A). These cells were infected with wild-type *C. trachomatis*, and the degree of colocalization of PI3K and Crk proteins with nascent inclusions was determined by indirect immunofluorescence. We did not detect any major differences in the efficiencies of colocalization of the bacteria with PI3K in CrkL-deficient HeLa cells and vice versa (Fig. 4B), suggesting that CrkL and PI3K may engage TepP independently of each other. Similarly, CrkI/II-deficient cell lines were not impaired for the recruitment of PI3K to early inclusions (Fig. 4B).

Previous studies based on RNA interference (RNAi)-mediated gene silencing suggested that both PI3K and Crk proteins are important for the replication of *Chlamydia* (35, 36). We assessed the efficiency of the various edited HeLa cell lines to generate *C. trachomatis* CTL2 infectious particles. PI3K-deficient HeLa cells displayed moderate (~50%) defects in the generation of infectious EBs at 48 hpi. CrkL- and CrkI/II-deficient lines displayed only mild (<25%) defects (Fig. 4C). However, we did not observe differences in the generation of EBs between wild-type and TepP-deficient *Chlamydia* among the various knockout (KO) HeLa cell lines, suggesting that the participation of PI3K and Crk proteins in promoting *Chlamydia* replication, at least in HeLa cells, occurs independently of their interactions with TepP.

### PI3K modulates TepP-dependent type I IFN responses.

**FIG 5:**
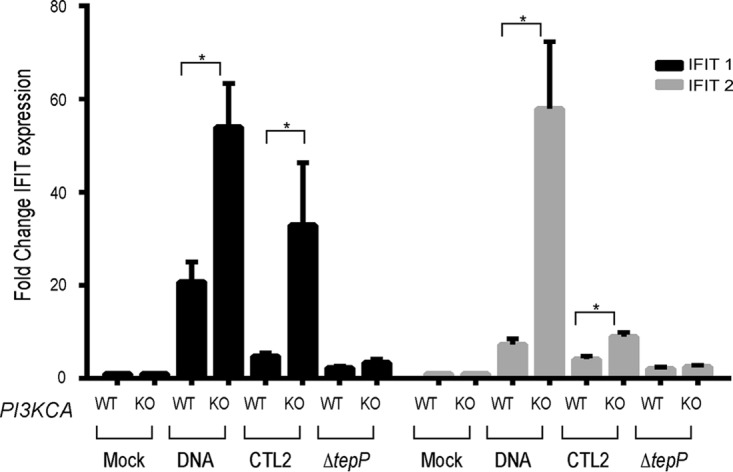
PI3K modulates TepP-dependent type I IFN responses. HeLa-Cas and *PI3KCA* KO cells were infected with CTL2 or *ΔtepP* mutants, and *IFIT1* (black bars) or *IFIT2* (gray bars) expression was assessed at 16 hpi by quantitative RT-PCR. Results from WT and DNA samples for both *IFIT1* and *IFIT2* are significantly different between HeLa-Cas and *PI3KCA* knockout cells (KO) (*P* value = <0.001, log-transformed ANOVA with *a priori* contrasts). Fold change values were calculated compared to mock-infected isolates. DNA transfections were included as controls for the induction of IFIT genes. The difference between HeLa-Cas and *PI3KCA* KO cell responses to DNA transfection or CTL2 infection was statistically significant (*P* value = <0.01 [Student’s *t* test]).

PI3K and CrkL participate in the activation of immune signaling (32, 37, 38). CrkL can interact with STAT5 to regulate the translocation of this transcription factor to the nucleus and the expression of type I interferon-induced genes (32, 39). We tested if PI3K and Crk adaptors were required for the TepP-dependent induction of *IFIT* genes. PI3K-, CrkL-, and Crk I/II-deficient HeLa cells were infected with either wild-type or *ΔtepP C. trachomatis*, and *IFIT1* and *IFIT2* expression was assessed at 16 h postinfection by quantitative PCR (qPCR). CrkI/II- and CrkL-deficient HeLa cells did not display any significant differences in their responses to either wild-type or mutant *Chlamydia*. In contrast, PI3K-deficient cell lines displayed an exaggerated *IFIT* response to wild-type bacteria (Fig. 5). These cell lines also displayed a stronger response to cytoplasmic DNA, suggesting a broader role for PI3K activity in modulating responses to cytosolic nucleic acids. Similar observations were made in HeLa cells treated with PI3K inhibitor LY294002 in response to both DNA and *Chlamydia* infection (Fig. S2). Overall, these observations imply that one of the functions of p110α at nascent inclusions is to dampen type I IFN responses to TepP-expressing *Chlamydia*.

10.1128/mSphere.00207-17.3 FIG S2  Treatment with the PI3K inhibitor LY294002 increased *IFIT* gene expression upon infection. *IFIT* expression is induced in HeLa-Cas upon infection with CTL2 for 16 h or upon DNA transfection. Treatment with LY294002 increased IFIT expression upon DNA transfection or infection with CTL2 but not after infection with Δ*tepP*::*bla* mutants. Fold change is calculated compared to mock-infected isolates. Results from WT and DNA samples for both *IFIT1* and *IFIT2* were significantly different between HeLa-Cas and *PI3KCA* KO (*P* value = <0.001 [log-transformed ANOVA with *a priori* contrasts]). Download FIG S2, TIF file, 1.3 MB. Copyright © 2017 Carpenter et al. 2017 Carpenter et al. This content is distributed under the terms of the Creative Commons Attribution 4.0 International license.

### TepP increases PI3K activity on early inclusions.

**FIG 6:**
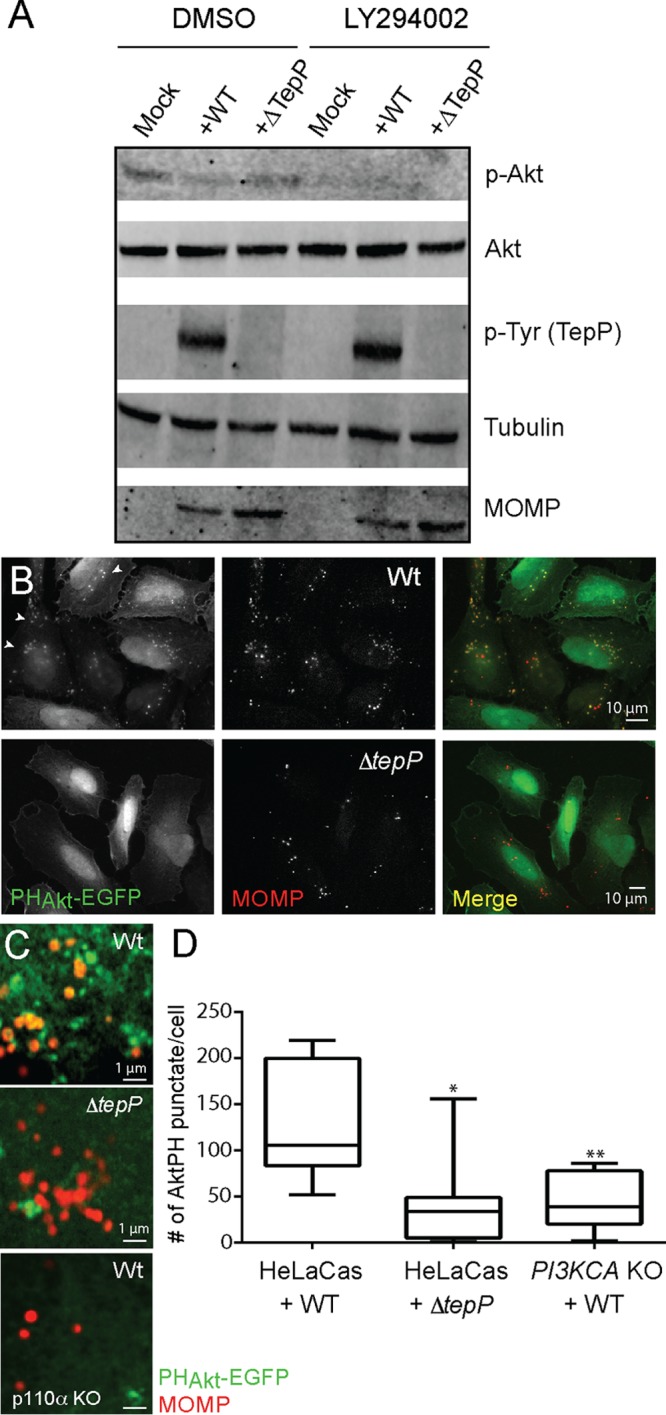
TepP activates PI3K at early inclusions. (A) Immunoblot analysis of HeLa-Cas cells infected with CTL2 or *ΔtepP*::*bla C. trachomatis* for 4 h indicated that phospho-Akt levels were not significantly altered by TepP. (B to D) Accumulation of PIP3-positive puncta, as assessed by the recruitment of PH_Akt_-EGFP, in HeLa cells infected with CTL2 but not Δ*tepP*::*bla C. trachomatis*. (B) HeLa cells were transfected with PH_Akt_-EGFP for 24 h, infected with the indicated *C. trachomatis* strains for 4 h, fixed, and immunostained with anti-MOMP antibodies. (C) Closeup image of clusters of internalized *C. trachomatis* displaying association with PIP3 (PH_Akt_-EGFP positive). Levels of bacterium-associated PH_Akt_-EGFP intracellular puncta were significantly reduced in CTL2-infected *PI3KCA* KO HeLa cells. (D) Quantification of PIP3-positive puncta in cells infected with CTL2 or Δ*tepP*::*bla C. trachomatis* was performed on a per-image basis with 7 to 10 fields in total. Note that punctum formation required the p110α subunit of PI3K. Significance was determined by Student’s *t* test (*, *P* < 0.01; **, *P* < 0.001).

In canonical PI3K/Akt signaling, activation of receptor tyrosine kinase (RTK) at the plasma membrane leads to the recruitment of PI3K and phosphorylation of phosphoinositide-(4,5)-bisphosphate (PIP2) to generate phosphoinositide-(3,5)-trisphosphate (PIP3) (21). Akt is then recruited to PIP3-enriched sites at the plasma membrane via its PH domain, leading to Akt phosphorylation and activation (21, 40). Activated Akt is an important regulator of multiple prosurvival signals (21, 27, 41). Upon *C. trachomatis* infection of HeLa cells, phosphorylation of Akt at Ser473 occurs with a bimodal distribution with early (before h 1) and late (after h 12) peaks of activation (42). We next tested if TepP contributed to the activation of PI3K and Akt. HeLa cells were infected with either wild-type or *ΔtepP C. trachomatis*, and the levels of Akt activation were assessed by immunoblot analysis with anti phospho-Akt antibodies. We observed low levels of p-Akt levels at 4 h postinfection, and this activity was dependent on PI3K activity as the signal was blocked by preincubation with the LY294002 inhibitor (Fig. 6A). Infection with *ΔtepP* bacteria did not result in significantly altered pAkt levels compared to infection with wild-type *C. trachomatis*, suggesting that PI3K activation at the plasma membrane is not overtly affected by TepP. The activation of Akt during *Chlamydia* infection was mostly mediated by the p110α isoform, as no phospho-Akt signal was detectable in p110α knockout (KO) cells (data not shown).

Although Akt phosphorylation is a common readout of increased activation of class I PI3Ks, additional cofactors such as PDK1 are required to phosphorylate Akt (21, 43). The most direct method of measurement of PI3K activity is by analysis of the generation of PIP3. To assess the levels and spatial distribution of PIP3 pools in infected cells, we transfected cells with constructs expressing enhanced green fluorescent protein (EGFP) fusions to the PH domains of Btk and Akt specifically binding to PIP3 (44). We observed an accumulation of PIP3-positive intracellular puncta in cells infected with wild-type but not *ΔtepP C. trachomatis* (Fig. 6C and D). These puncta were observed in the vicinity of nascent inclusions but were not always completely overlapping (Fig. 6D) and were not observed in PI3K-deficient cells, suggesting that TepP mediates the localized formation of PIP3 though the activation of PI3K activity (Fig. 6D).

## DISCUSSION

The process of *Chlamydia* entry into cells and of establishment of a nascent inclusion is mediated by the controlled reorganization of the cytoskeleton and membranes by effector proteins. We determined that the early secreted effector protein TepP recruits the adaptor protein CrkL and the lipid kinase PI3K, leading to the localized synthesis of PIP3 on early inclusions (Fig. 1, 2, and 6). Based on TepP’s ability to stably recruit PI3K, CrkL, and CrkI/II proteins and on the role that Crk proteins play as adaptors linking receptors to signaling outputs, we hypothesize that TepP acts as a scaffold for the localized reprogramming of signaling proteins early during infection.

We generated a strain with a *bla* gene insertion in *tepP* in a clean genetic background to assess the phenotypic difference between wild-type *C. trachomatis* and strains deficient only for the expression of TepP. All TepP-dependent phenotypes described previously in a chemically derived *tepP* mutant and its complemented counterparts (6) were validated using this new deletion strain and further allowed us to reveal a contribution of TepP to bacterial replication in A2EN cells. A2EN cells were recently derived from cervical tissue explants and maintain phenotypic and functional characteristics of endocervix epithelial cells, including cell polarization, mucus secretion, and expression of antimicrobial peptides in response to microbial Toll-like receptor (TLR) agonists (45). Unfortunately, because these cells are not readily transfectable, in some instances we had to rely on alternative cell lines to test the role of host factors in TepP-mediated activities. In all instances, we found our results obtained with genetically altered cell lines (HeLa and MEFs) and inhibitor studies in A2EN cells to be internally consistent. The only exception was PI3K inhibitor LY294002, which we did not find to be effective in A2EN cells at levels that did not induce cytotoxicity (data not shown).

Previous genome-wide RNAi screens aimed at identifying host factors important for *Chlamydia* proliferation identified PI3K and Crk proteins as important for *Chlamydia* entry and replication (35, 46). These growth defects were confirmed in HeLa cells in which p110α and Crk genes had been inactivated through CRISPR/Cas9-mediated gene editing, with p110α-deficient lines showing the greatest impairment (Fig. 4). These host proteins may contribute to pathogen replication by facilitating entry, activation of pathways important for nutrient acquisition, and/or inactivation of cell defense mechanisms. However, although TepP is required for the recruitment of CrkL and PI3K to nascent inclusions early in infection, their contribution to enhancing bacterial replication, at least in HeLa cells, does not appear to have occurred through interaction with TepP as *ΔtepP* mutants replicated to levels similar to those seen with wild-type *C. trachomatis* in cells where Crk and PI3K had been inactivated (Fig. 4).

We expected that most host factors would specifically associate with phosphorylated versions of TepP. This appears not to be the case, as the interaction between TepP and PI3K and between TepP and CrkL, at least within the limits of our assays, was not affected by Src-mediated phosphorylation of TepP or by mutation of the three major tyrosines that are phosphorylated (Fig. 3; see also Fig. S1 in the supplemental material). It is possible that other tyrosine kinases or additional tyrosine phosphorylation sites may independently contribute to the recruitment of PI3K, Crk adaptors, and/or other SH2 and PTB domain-containing proteins. Because Crk proteins bridge phosphorylated RTKs to p85 to activate PI3K (33), we also expected that the binding of the p85/p110 complex to TepP would be mediated by CrkL. However, we found that recruitment of these proteins to early inclusions occurred independently of each other. The influenza virus protein NS1 independently binds to p85 and CrkL to activate PI3K activity (47, 48). TepP could be performing a similar function by assembling a tripartite Crk-TepP-PI3K complex that potentiates kinase activity as exemplified by the marked accumulation of PIP3-positive vesicles at the periphery of early inclusions. Alternatively, because the presence of TepP increased the efficiency of PI3K coimmunoprecipitation (co-IP) with CrkL (Fig. 1C), the possibility exists that Crk proteins further escalate the recruitment of additional p85/p110 to early inclusions and to enhancement of PI3K activity. We note that the activation of PI3K activity by TepP at intracellular sites did not lead to increased phosphorylation of Akt, suggesting that the activity of this kinase is uncoupled from events that may occur at the plasma membrane. In general, while PI3K and Crk proteins have been shown to be required for optimal *Chlamydia* replication (35, 46), this does not necessarily appear to have been linked to TepP, even though this effector appears to be a major contributor to the recruitment of these proteins to nascent inclusions. It remains to be determined whether pleotropic effects resulting from the disruption of these host factors are indirectly responsible for their impact on bacterial fitness.

The prolonged recruitment of PI3K and Crk proteins on early inclusions that have migrated to perinuclear regions of infected cells correlates with the kinetics of TepP translocation after EB entry into cells (6). We predict that this process of recruitment and activation is independent of events that occur during the initial stages of infection (<1 h) where engagement of RTKs like the epidermal growth factor receptor (EGFR) (49), EphrRA2 (50), and platelet-derived growth factor receptor (PDGFR) (35) and/or tyrosine-phosphorylated Tarp may transiently recruit Crk proteins and PI3K to entry sites. The consequence of PI3K activation by TepP is the localized production of PIP3 at and in the vicinity of internalized bacteria (Fig. 6), which we predict leads to the recruitment of specialized PH domain-containing proteins that further influence downstream signaling and/or cytoskeletal and membrane remodeling events. It remains to be determined how TepP orchestrates PI3K activity and Crk and GSK-3 binding with the potential recruitment of additional factors. But the ultimate consequence of a failure to recruit all (or a subset) of those factors is a decrease in *Chlamydia* fitness in cervical epithelial cells and a decrease in the transcription of genes associated with type I IFN responses.

Although PI3K and Crk proteins are important for full bacterial replication, as assessed by reduced growth in cells where these factors have been deleted, their role in promoting *Chlamydia* replication does not appear to be mediated by TepP, at least in HeLa cells. PI3K, but not Crk protein(s), however, does appear to influence the magnitude of IFN responses to TepP-deficient bacteria (Fig. 5). PI3K knockout HeLa cells showed a >5-fold increase in IFIT expression upon infection with TepP-expressing bacteria. PI3K regulates innate immunity in a variety of ways (48, 51), and inhibition of PI3K in dendritic cells enhances IFN-β transcription (52). It is possible that the absence of TepP increases the availability of microbial products from early inclusions to innate immune sensors or that PI3K activity modulates the function of sensors, which is consistent with the finding that basal expression of *IFIT* genes increases 2-fold to 3-fold in response to the presence of cytosolic DNA (Fig. 5).

Overall, our findings begin to delineate important molecular components that are specifically assembled on *C. trachomatis* early inclusions that are ultimately important for fitness of the pathogen and for dampening of innate immune signaling in response to infection. The identity of the molecular players downstream of increased PI3K at intracellular sites and how they modulate the expression of immunity genes and bacterial survival in cervical epithelial cell remain to be determined.

## MATERIALS AND METHODS

### Cell lines, bacterial strains, and reagents.

*Chlamydia trachomatis* serotype LGV-L2, strain 434/Bu(CTL2), and subsequently derived strains (*C. trachomatis* Δ*tepP*::*bla*, CTL2-M062G1 [*tepP*^Q103^*], CTL2-M062G1 [pVec] [6], and CTL2-M062G1 [pTepP] [6]) were propagated in Vero cells maintained in Dulbecco’s modified Eagle medium (Sigma-Aldrich, St. Louis, MO, USA) supplemented with 10% fetal bovine serum (Mediatech, Manassas, VA). Vero, HeLa (and associated Cas9 variants), and HEK293T cells were propagated in Dulbecco’s modified Eagle medium (Sigma-Aldrich, St. Louis, MO, USA) supplemented with 10% fetal bovine serum (Mediatech, Manassas, VA) (see also Text S1 in the supplemental material). A2EN cells were propagated in keratinocyte–serum-free medium (SFM) (Gibco, Life Technologies, Inc., Grand Island, NY, USA) supplemented with 10% fetal bovine serum (FBS), 0.5 ng/ml human recombinant epidermal growth factor, and 50 μg/ml bovine pituitary extract. EBs were purified by density gradient centrifugation using Omnipaque 350 (GE Healthcare, Princeton, NJ) as previously described (18). PH-Akt-GFP (Addgene plasmid 51465 [53]) was a gift from Tamas Balla. Recombinant TepP was expressed in *Escherichia coli* strain BL21(DE3) as previously described (6). All reagents used were analytical grade. Transfection reagent Lipofectamine 2000 was purchased from Invitrogen. Opti-MEM serum-free media and rat tail collagen I were purchased from Gibco.

10.1128/mSphere.00207-17.1 TEXT S1  Supplemental materials and methods. Download TEXT S1, PDF file, 0.2 MB. Copyright © 2017 Carpenter et al. 2017 Carpenter et al. This content is distributed under the terms of the Creative Commons Attribution 4.0 International license.

### Generation of Δ*tepP*::*bla C. trachomatis* strains.

Recombinant *C. trachomatis* strains were generated through the use of a modified version of a CaCl_2_ DNA transformation protocol that was previously described (54). A TargeTron targeted gene disruption system (Sigma-Aldrich) was used to insert a *bla* cassette at the *tepP* locus between amino acids 821 and 822 (ATCTCTCTGGATAATACAACGTCTGAGAAA-intron-TTGCTCATGTCCAGC) in CTL2. Plaque-purified recombinants were expanded in Vero cells, and proper targeting was confirmed by PCR with primers flanking the insertion site and by Western blot analysis with anti-TepP antibodies (Fig. 2A). Whole-genome sequencing of this strain indicated that no additional mutations were present.

### IFU burst assay.

A2EN and HeLa-Cas cells were seeded onto 96-well plates (15,000 cells/well), and 24 h later, cells in each well were infected with the indicated strains (three biological replicates per time point) at a multiplicity of infection (MOI) of approximately 0.5. The number of input inclusion-forming units (IFU) was assessed at 24 h by fixing infected cells with 100% methanol (EMD Millipore) for 10 min on ice and immunostaining with anti-CTL2 sera and Alexa Fluor-conjugated secondary antibodies (Invitrogen Life Technologies, Inc., Carlsbad, CA). Images were acquired in a Zeiss Axioskop 2 upright epifluorescence microscope using at least 3 different fields per replicate, and the numbers of inclusions were counted. To assess bacterial replication and infectivity, infections were allowed to proceed for 48 h, cell were lysed by hypotonic lysis, and IFUs were calculated on monolayers of Vero cells seeded onto 96-well plates as described above. The total number of infected progeny released (output) was divided by the total number of input infectious particles. All statistical analysis was performed using GraphPad Prism.

### *In vitro* kinase assays.

His-TepP was purified from *E. coli* strain BL21 and grown in LB broth with plasmid induction and IPTG (isopropyl-β-d-thiogalactopyranoside) (Sigma; catalog no. 367-93-1) at a concentration of 0.5 mM for 4 h at 37°C. Cells were lysed with lysis buffer (5 M NaCl, 10% Triton X-100, 1 M imidazole, 250 mM phenylmethylsulfonyl fluoride [PMSF], 20 mM phosphate) supplemented with lysozyme (Thermo Fisher Scientific; catalog no. 89833) with sonication to reduce viscosity and centrifugation to pellet cell debris. Purification of His-TepP was performed using nickel resin (Thermo Fisher Scientific; catalog no. 88221) and end-over-end incubation at 4°C for 1 h. The resin was washed three times with lysis buffer and was subsequently washed three times with *in vitro* kinase buffer (1 M HEPES, 2 M MgCl_2_, 250 mM KCl, 1ATP, PMSF, Halt protease inhibitor cocktail [Thermo Fisher Scientific; catalog no. 78430], and complete phosphatase inhibitor cocktail [Sigma; catalog no. 4693159001]). Bound His-TepP was then incubated with whole-cell lysates (lysed with *in vitro* kinase buffer and light sonication) from Vero, MEF, SYF, SYF+Src, Abl/Arg−/− MEFs, and its complemented counterparts for 1 h with end-over-end mixing at 4°C. Bound TepP was then washed five times with *in vitro* kinase buffer without ATP. His-TepP was eluted from the nickel resin by incubation with Laemmli sample buffer and boiling. Levels of phosphorylation were assessed by immunoblot staining with anti-phosphotyrosine (Cell Signaling; catalog no. 9411), and TepP levels were assessed by immunoblot staining with anti-TepP (6).

### Indirect immunofluorescence microscopy.

A2EN cells were seeded at a density of approximately 8 × 10^4^ cells/well on glass coverslips precoated with 30 μg/ml collagen–20 mM acetic acid (Spectrum) for 5 min and rinsed twice with fresh media. HeLa and MEF cells were seeded at a density of 5 × 10^4^ cells/well on glass coverslips. The following day, CTL2 EBs were added at the indicated MOI (20 or 100). Infections in A2EN cells were synchronized by centrifugation at 500 × *g* for 5 min at 10°C, whereas infections in HeLa cells and MEFs were synchronized by centrifugation at 1,000 × *g* for 20 min at 10°C.The medium was replaced, and the infected cells were transferred to a humidified incubator and maintained at 37°C and 5% CO_2_. Coverslips were fixed with ice-cold 100% methanol for 15 min, rehydrated with phosphate-buffered saline (PBS) (using three 5-min washes), and blocked with 2% bovine serum albumin (BSA)–PBS for 20 min.

To monitor the levels of PIP3 on early inclusions, HeLa cells were transfected with a plasmid encoding PH-Akt-GFP. After 24 h, cells were infected with strain CTL2 or CTL2 *ΔtepP*::*bla* for 4 h, rinsed twice with PBS, and fixed with 1.5% formaldehyde (Sigma)–PBS for 20 min. Cells were quenched with 0.25% ammonium chloride (Sigma) and permeabilized/blocked with 2% BSA containing 0.1% saponin for 30 min.

Rabbit antibodies against TepP (1:50) (6), p110α (Cell Signaling; catalog no. 9411) (1:100), Slc1 (1:500) (6), Crk (BD Transduction Laboratories; catalog no. 610035, clone 22) (1:50), CrkL (Thermo Fisher Scientific; catalog no. PA5-28622) (1:100), and a mouse antibody against major outer membrane protein (MOMP) (Santa Cruz; catalog no. sc-57678) (1:500) were diluted in PBS containing 2% BSA. Secondary antibodies, including goat anti-mouse (H+L) Alexa Fluor 488 and Alexa Fluor 555 (Thermo Fisher Scientific; catalog no. A11001 and A21422) and goat-anti-rabbit (H+L) Alexa Fluor 488 and Alexa Fluor 555 (Thermo Fisher Scientific; catalog no. A11034 and A21428) antibodies, were diluted in PBS containing 2% BSA. Hoechst stain (Invitrogen; catalog no. H3570) was diluted in PBS and incubated on the cells for 10 min. Coverslips were mounted on slides using Fluorsave mounting media (CalBiochem).

Cells were imaged using a confocal laser scanning microscope (LSM 880; Zeiss) equipped an Airyscan detector (Hamamatsu) and diode (405 nm) and Aragon ion (488-nm), double solid-state (561-nm), and helium-neon (633-nm) lasers. Images were acquired using a 63×/1.4 numerical aperture (NA) oil objective (Zeiss) and deconvolved using automatic Airyscan processing in Zen software (Zeiss). In Fig. 6B, PH-Akt-GFP-expressing HeLa cells were imaged using a Zeiss AxioObserver Z.1 wide-field microscope equipped with a motorized stage and a camera (AxioCam MRm; Zeiss). Image were acquired using a 63×/1.4 NA oil objective (Zeiss) and AxioVision 4.1 software.

### Image processing.

All images were processed using ImageJ open source software (ImageJ). Linear adjustments were made for all images, and maximum projections from Z-stacks are portrayed. Quantification of recruitment of proteins to the nascent inclusion was performed in ImageJ using JACoP (just another colocalization plugin) on a per-cell basis on each z-slice of each image. Percent colocalization was calculated using distance-based colocalization and the percentages of positive threshold pixels of MOMP that associated with threshold pixels of the p110α, CrkL, or GSK-3β proteins. Thresholds were set to eliminate background signals in both the MOMP- and TepP-associated protein channels. Quantification of PIP3-positive puncta (PH-Akt-GFP) was performed by using the *Analyze Particles* function in ImageJ. Strict thresholding parameters were applied to reduce the number of false positives.

### Immunoprecipitations.

Antibodies against CrkL (PA5-28622), p110α (Cell Signaling Technology, Inc.; catalog no. 4249S [α]), and FLAG (conjugated to magnetic beads [Sigma-Aldrich, St. Louis, MO, USA; catalog no. M8823]) were incubated for 2 h at 4°C with total cell lysates derived from 6 × 10^6^ A2EN cells infected with the indicated *C. trachomatis* CTL2 strains at an MOI of 100. Infected cells were lysed in 750 μl of radioimmunoprecipitation assay (RIPA) buffer (Sigma; catalog no. R0278) supplemented with 1× EDTA free protease inhibitor cocktail (Roche, Basel, Switzerland) and Halt phosphatase inhibitor (Thermo Fisher Scientific; catalog no. 78428). After a 2-h period, CrkL and p110α samples were incubated for 1 h at 4°C with magnetic protein A-conjugated beads (SureBeads protein A magnetic beads [Bio-Rad; catalog no. 161-4011]). All magnetic bead slurries were then washed five times with 200 μl of lysis buffer, and bound proteins were eluted in Laemmli sample buffer and identified by Western blot analysis.

### Identification of TepP-FLAG interacting proteins.

Four 15-cm-diameter dishes of confluent human A2EN cells were infected either with CTL2-M062G1 (*tepP*^Q103^*) or with CTL2-M062G1 transformed with a plasmid expressing TepP-FLAG. Cells were infected at an MOI of 50 by rocking at 4°C for 30 min in HBSS (Hanks’ balanced salt solution) (Invitrogen Life Technologies, Inc., Carlsbad, CA), following by shifting to 37°C after addition of warm keratinocyte-SFM. After 4 h, cells were washed once with cold PBS and lysed on ice by scraping in lysis buffer (25 mM Tris, 150 mM NaCl, 1 mM EDTA, 1% NP-40, 5% glycerol; pH 7.4) supplemented with 1 mM phenylmethylsulfonyl fluoride (PMSF), 1× EDTA-free protease inhibitor cocktail (Roche, Basel, Switzerland), and Halt phosphatase inhibitor (Pierce, Rockford, IL). Cell debris was pelleted by centrifugation, and the supernatants were transferred to a new tube. Around 100 μl of anti-FLAG M2 magnetic beads (M8823) was added to each tube and mixed by constant rotation at 4°C for 4 h. After 5 washes with lysis buffer, the bound proteins were eluted with Pierce elution buffer (Pierce, Rockford, IL) (pH 2.8).

Proteins were concentrated into ammonium bicarbonate using 0.5-ml Amicon 10-molecular-weight-cutoff (MWCO) filters. After concentration, samples were separated on a NuPAGE 4% to 12% bis-Tris SDS-PAGE gel (Thermo Fisher Scientific). After staining with Novex colloidal Coomassie stain (Thermo Fisher Scientific) was performed, three bands were isolated for each sample, covering the approximate molecular mass range of 20 kDa to 150 kDa. The three bands per sample were excised and destained, and the proteins in the bands were digested with trypsin according to the in-gel tryptic digestion protocol (https://genome.duke.edu/sites/genome.duke.edu/files/In-gelDigestionProtocolrevised.pdf). Briefly, bands were destained with 1:1 MeCN/water, reduced with 10 mM dithiothreitol, alkylated with 20 mM iodoacetamide, and then dehydrated in MeCN and swelled in 50 mM ammonium bicarbonate containing 10 ng/ml trypsin. Digestion was carried out overnight at 37°C, and digestion was quenched and peptides were extracted using 0.1% (vol/vol) trifluoroacetic acid (TFA)–1:1 MeCN/water. Samples were dried and reconstituted in 10 ml 1/2/97 (vol/vol/vol) TFA/MeCN/water for mass spectrometry analysis. Liquid chromatography-tandem mass spectrometry (LC-MS/MS) for peptide sequencing was performed on a nanoAcquity ultraperformance liquid chromatography (UPLC) system coupled to a Synapt G2 high-definition mass spectrometry (HDMS) system (Waters Corporation). Raw data were processed in Mascot Distiller (Matrix Sciences), and Mascot Server v2.5 (Matrix Sciences) was used for database searching. A custom *.Fasta database was constructed by combining the curated human proteome (uniprot.org) and the *Chlamydia trachomatis* L2/434/Bu genome along with common laboratory contaminants. Database searching used 10-ppm precursors and 0.04-Da product ion tolerance, fixed carbamidomethylation (Cys), and various levels of oxidization (Met) and deamidation (nonquantitative [NQ]). Database search results were curated in Scaffold (Proteome Software) to a 0.38% peptide false-discovery rate (FDR) and 0.5% protein FDR, using decoy database searching and the PeptideProphet algorithm.

### RNA isolation and RT-qPCR.

RNA was collected from 3 wells of a 6-well plate of infected cells at 16 hpi using an RNeasy Plus minikit (Qiagen; catalog no. 74134). A2EN, HeLa-Cas9, and HeLa-Cas9 KO cell lines were seeded 24 h prior to infection at a density of approximately 0.8 × 10^6^ cells per well. The cells were then infected with gradient-purified EBs of either CTL2 or CTL2 *ΔtepP*::*bla* strains at an MOI of 10. As controls, cells were also subjected to mock infection or transfected with 5 µg of a linearized bacterial plasmid (pET-24d), using a jetPRIME transfection system (Polyplus transfection; VWR catalog no. 89129-922). Real-time quantitative PCR (RT-qPCR) was conducted using a Power SYBR green RNA-to-C_T_ 1-Step kit (Thermo Fisher Scientific). To assess the levels of *IFIT1-2* gene expression (the primers that were used are described in reference 6), relative expression levels were determined according to the comparative threshold cycle (*C*_*T*_) method (55) using β-actin mRNA as the reference for normalization. Results of mock, DNA, wild-type strain, and strain *ΔtepP* treatments were analyzed via log-transformed analysis of variance (ANOVA) (aov function, “stats” package) in R (56) with *a priori* contrasts between the HeLa-Cas9 and *PI3KCA* KO cell lines. Both the WT and DNA results showed significant (*P* < 0.001) differences between the HeLa-Cas9 and *PI3KCA* KO cell lines, while there were no significant differences in the results from mock treatment or strain *ΔtepP* treatment between HeLa-Cas9 and *PI3KCA* KO mutant cells. This was observed for both *IFIT1* and *IFIT2*.

### Generation of CRISPR/Cas9 KO cells.

HeLa KO cell lines were generated by stable integration of vectors expressing single guide RNA (sgRNA) into Cas9-expressing HeLa cells. The following sgRNAs were used: GAGGACATGGTGTTGGACCG (*CRKL*), GACTTTAGAATGCCTCCGTG (*PI3KCA*), and GGGGAGGTTGAGTCGGCAGG (*CRK*). To generate transducing viruses, HEK293T cells were cotransfected with vectors pasPAX2 and pCMV-VSV-G and the sgRNA in lentiGuide-Puro (Addgene) (three times at 12-h intervals). Supernatants containing virus were collected, filter sterilized (0.2 μm), and incubated with HeLa-Cas9 cells (Duke Functional Genomics Core facility) at 48 and 72 h. At 24 h after the last round of viral infections, the medium was replaced with fresh media containing 5 μg of puromycin/ml to select for stably transduced cells. Selection was continued until all cells in mock-infected cells were dead. Cells were then clonally isolated by limiting dilution, and each cell line was assessed for protein expression by immunoblot analysis.
