# Supplementary figures and images for: The Effector TepP Mediates Recruitment and Activation of Phosphoinositide 3-Kinase on Early Chlamydia trachomatis Vacuoles
Source: mSphere. 2017 Jul 19;2(4):e00207-17. doi: 10.1128/mSphere.00207-17 (PMC5518268; doi:10.1128/mSphere.00207-17)

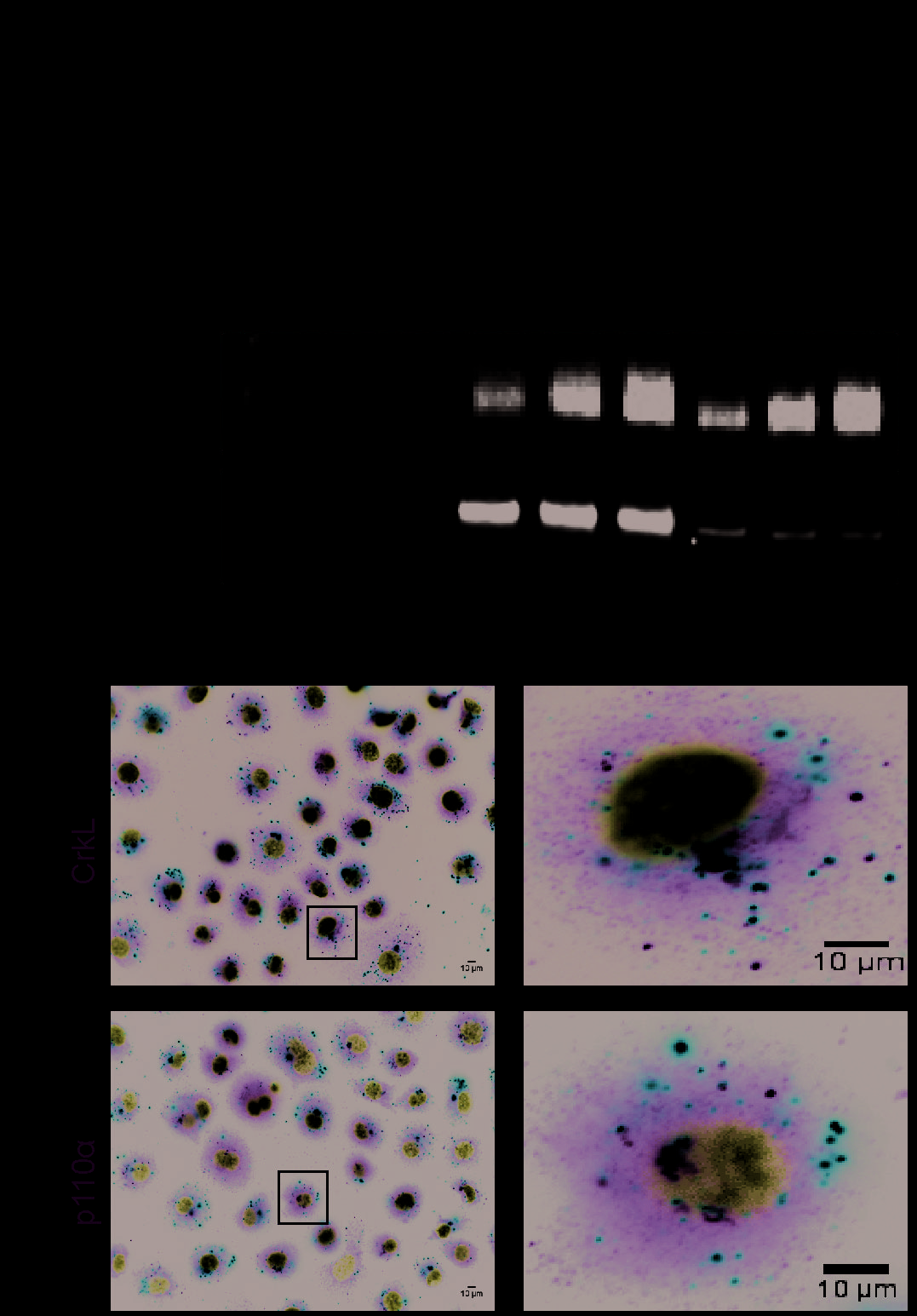

Supplement: FIG S1 [file sph004172323sf2.tif]

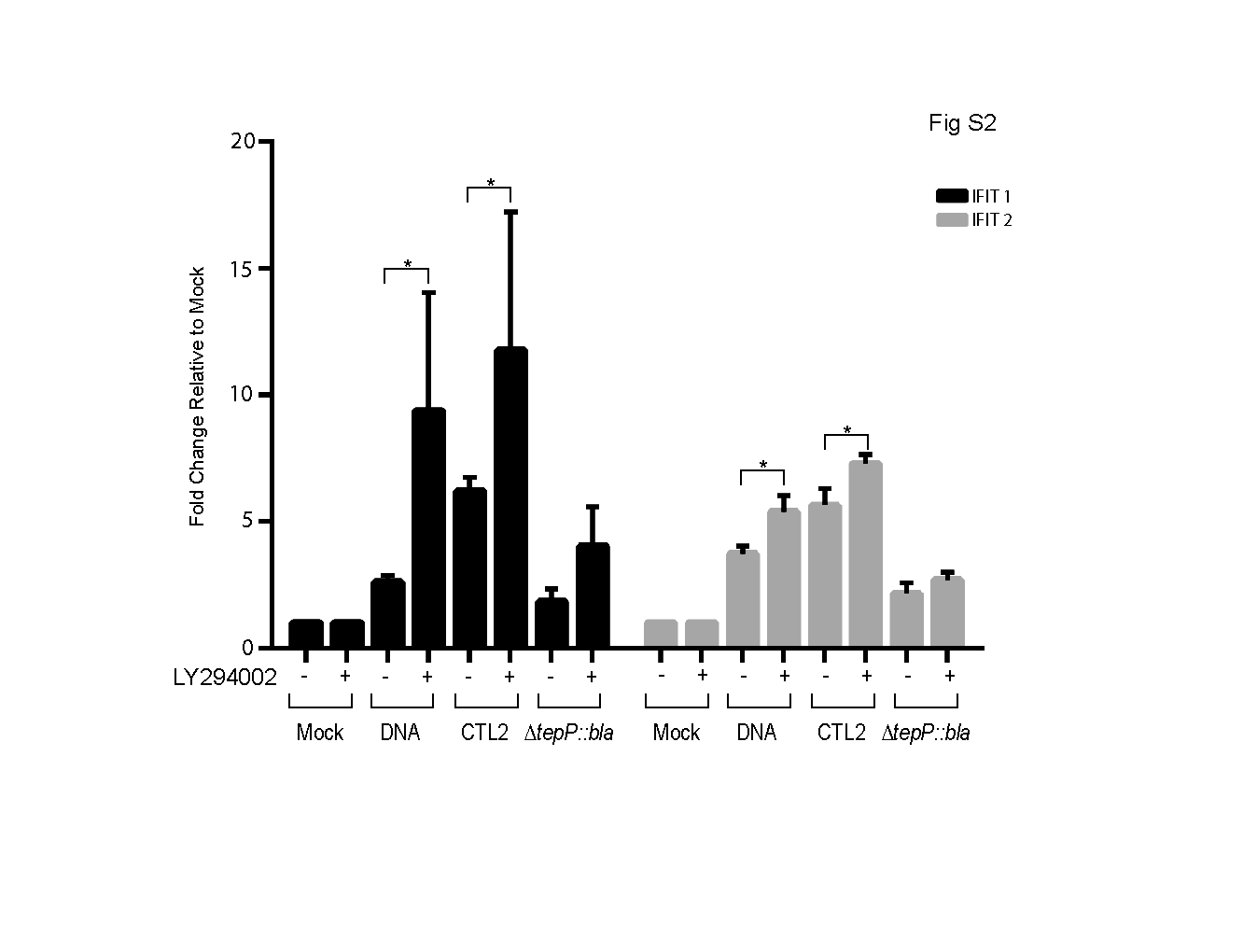

Supplement: FIG S2 [file sph004172323sf3.tif]
