# Supplementary material for: The Effector TepP Mediates Recruitment and Activation of Phosphoinositide 3-Kinase on Early Chlamydia trachomatis Vacuoles
Source: mSphere. 2017 Jul 19;2(4):e00207-17. doi: 10.1128/mSphere.00207-17 (PMC5518268; doi:10.1128/mSphere.00207-17)
Supplement: TEXT S1 [file sph004172323s4.pdf]

Supplemental Materials and Methods

Generation of TepP point mutants

TepP tyrosine phosphorylation mutants were generated using site directed mutagenesis with the Q5 site-directed mutagenesis kit (New England Biolabs, E0554S). Each tyrosine site (Y43, Y496, and Y504) was mutated individually to phenylalanines in the +pTepP vector (1). Each version of the plasmid was transformed into *C. trachomatis* L2-M062G1 (TepP deficient strain) using a modified version of the $CaCl_2$ transformation protocol previously described (2). Expression and translocation of TepP variants was verified with FLAG anti-bodies in immunoblot and immunofluorescence staining.

LY249002 Inhibitor treatment, RNA isolation, and RT-qPCR

HeLa-Cas cells were seeded 24 hours prior to infection at a density of approximately 0.8×10^6 cells per well. Cells were incubated with DMSO (solvent only control) or the PI3K kinase inhibitor LY294002 (Cell Signaling, 9901S) at a concentration of 20µM/mL for 1 hour prior to infection. Cells were then infected with gradient purified CTL2 or CTL2 *ΔtepP::bla* EBs at an MOI of 10. As a control, cells were also mock infected or transfected with 5ug of a linearized bacterial plasmid (pET-24d) using jetPRIME transfection system (Polyplus-transfection, VWR 89129-922). RNA was collected from three wells of a 6-well plate at 16 hpi, DMSO or inhibitor treatment was maintained throughout infection. RT-qPCR was conducted using the Power SYBR Green RNA-to-CT™ 1-Step Kit (Thermo-Fisher Scientific). To assess differential gene expression of the IFITs (primers described in reference 1) relative expression levels were determined according to the comparative CT method, using β-actin mRNA as reference for normalization. Mock, DNA, WT, and *ΔtepP::bla* infections were analyzed via log-transformed anova (aov function, "stats" package) in R (3) with a priori contrasts between DMSO and LY294002 treated HeLa-Cas cells. WT and DNA both showed significant ($p<0.0001$) differences between DMSO and LY294002 treated HeLa-Cas cells while there were no significant differences for Mock, and small difference ($p<0.05$) during *ΔtepP::bla* infection between DMSO and LY294002 treated HeLa-Cas cells. This was observed for both *IFIT1* and*IFIT2*.

1. **Chen YS, Bastidas RJ, Saka HA, Carpenter VK, Richards KL, Plano GV, Valdivia RH.** 2014. The *Chlamydia trachomatis* type III secretion chaperone Slc1 engages multiple early effectors, including TepP, a tyrosine-phosphorylated protein required for the recruitment of CrkI-II to nascent inclusions and innate immune signaling. PLoS Pathog **10:**e1003954. https://doi.org/10.1371/journal.ppat.1003954.
2. **Wang Y, Kahane S, Cutcliffe LT, Skilton RJ, Lambden PR, Clarke IN.** 2011. Development of a transformation system for *Chlamydia trachomatis*: restoration of glycogen biosynthesis by acquisition of a plasmid shuttle vector. PLoS Pathog **7:**e1002258. https://doi.org/10.1371/journal.ppat.1002258.
3. **R Core Team.** 2016. R: A language and environment for statistical computing, v3.2.2. R Foundation for Statistical Computing, Vienna, Austria. https://www.R-project.org/.
